# Differential Effects of Exercise Programs on Neuregulin 4, Body Composition and Cardiometabolic Risk Factors in Men With Obesity

**DOI:** 10.3389/fphys.2021.797574

**Published:** 2022-02-07

**Authors:** Ayoub Saeidi, Sevda R. Shishvan, Mohammad Soltani, Fatemeh Tarazi, Patricia K. Doyle-Baker, Shahnaz Shahrbanian, Shirin S. Mollabashi, Nikoo Khosravi, Ismail Laher, Terence A. Moriarty, Kelly E. Johnson, Trisha A. VanDusseldorp, Hassane Zouhal

**Affiliations:** ^1^Department of Physical Education and Sport Sciences, Faculty of Humanities and Social Sciences, University of Kurdistan, Sanandaj, Iran; ^2^Department of Physical Education and Sport Science, Islamic Azad University, Tehran, Iran; ^3^Department of Biological Sciences in Sport, Faculty of Sports Sciences and Health, Shahid Beheshti University, Tehran, Iran; ^4^Department of Exercise Physiology, Faculty of Physical Education, Alzahra University, Tehran, Iran; ^5^Human Performance Lab, Faculty of Kinesiology, University of Calgary, Calgary, AB, Canada; ^6^Department of Sports Sciences, Faculty of Humanities, Tarbiat Modares University, Tehran, Iran; ^7^Faculty of Sport Sciences, Atatürk University, Erzurum, Turkey; ^8^Department of Anesthesiology, Pharmacology and Therapeutics, Faculty of Medicine, University of British Columbia, Vancouver, BC, Canada; ^9^Department of Exercise and Sport Science, Coastal Carolina University, Conway, SC, United States; ^10^Department of Exercise and Sport Science, Coastal Carolina University, Myrtle Beach, SC, United States; ^11^Department of Exercise & Sport Management, Kennesaw State University, Kennesaw, GA, United States; ^12^Laboratoire Mouvement, Sport, Santé – EA 1274, University Rennes, Rennes, France; ^13^Institut International des Sciences du Sport, Iroduer, France

**Keywords:** exercise, neuregulin 4 (Nrg4), obesity, HIIT (High Intensity Interval Training), resistance exercise and aerobic exercise

## Abstract

**Background:**

Neuregulin 4 (Nrg4) is an adipokine that is sensitive to energy expenditure and with a potential role in metabolic homeostasis and obesity. This study examined the effects of 12 weeks of three different exercise training protocols on Nrg4 levels, cardiometabolic risk factors, and body composition parameters in men with obesity.

**Methods:**

Sixty adult men with obesity (Mean ± SD; age: 27.60 ± 8.4 yrs.; height: 168.4 ± 2.6 cm; weight: 96.7 ± 7.2 kg) were randomly allocated into four equal (*n* = 15) groups: High- Intensity Interval Training (HIIT), Circuit Resistance Training (CRT), Moderate Intensity Continuous Training (MICT) or a control group. The HIIT protocol involved six bouts of 3-min high-intensity exercise (90% VO_2*peak*_) followed by 3-min low-intensity exercise (50% VO_2*peak*_). The CRT group performed three circuits of resistance training, where each circuit included 11 exercises at 20% of one-repetition maximum (1RM) and 70% of VO_2*peak*_, and with a work-to-rest ratio of 2:1 (40-s exercise and 20-s rest) and 60-s recovery between circuits. The MICT group performed 36 min of exercise at 70% of VO_2*peak*_. All measurements were taken 72 h before and after the first and last training sessions.

**Results:**

There were significant differences between the groups in fat-free mass (FFM), (effect size (ES): 0.78), fat mass (ES: 0.86), VO_2*peak*_ (ES: 0.59), high-density lipoprotein cholesterol (HDL-C) (ES: 0.83), low-density lipoprotein (LDL-C) (ES: 0.79), total cholesterol (TC) (ES: 0.90), triglyceride (TG) (ES: 0.52) glucose (ES: 0.39), insulin (ES: 0.61), HOM-IR (ES: 0.91) and Nrg4 (ES: 0.98) (*p* < 0.05). There were no significant changes in very-low-density lipoprotein cholesterol (VLDL-C) (ES: 0.13) levels, or body weights (ES: 0.51) (*p* > 0.05). Levels of Nrg4 were negatively correlated with LDL-C, TC, TG, VLDL-C, glucose, insulin, HOMA-IR (*p* < 0.05) and positively with HDL-C (*p* < 0.05).

**Conclusion:**

Our results suggest that HIIT and CRT protocols have greater effects than MICT protocol on Nrg4 levels, metabolic and cardiovascular risk factors, and body composition variables in men with obesity.

## Introduction

The global epidemic of obesity is associated with comorbidities such as cardiovascular diseases, type 2 diabetes, and dyslipidemia due to hypertrophy of adipose tissue (Weisberg et al., 2003). Adipocytes secrete various bioactive molecules, adipokines, and inflammatory factors such as visfatin, tumor necrosis factor-alpha (TNF-α), and neuregulin-4 (Nrg4; Blüher, 2019; Saeidi et al., 2020a). Some of these adipokines have detrimental effects, while others modulate glucose homeostasis, insulin resistance, lipid metabolism, and obesity-related diseases (Gumà et al., 2020; Saeidi et al., 2020a; Shanaki et al., 2020). Adipose tissue-derived Nrg4 is a signaling molecule enriched in brown adipose tissue (BAT), which targets the liver and is involved in metabolic homeostasis (Blüher, 2019; Tutunchi et al., 2020). Moreover, Nrg4 has an influential role in maintaining energy balance by having anti-lipogenic properties (Wang et al., 2014; Blüher, 2019). Additionally, Nrg4 is an adipokine that is sensitive to energy expenditure and body composition variables (Tutunchi et al., 2020) and the expression of Nrg4 mRNA decreases in obese mice. In overweight humans, the expression of Nrg4 in subcutaneous fat is negatively correlated with body mass index (BMI), body fat, and fatty liver (Wang et al., 2014; Ma et al., 2016; Chen Z. et al., 2017). Furthermore, Nrg4 mRNA expression levels are lower in patients with impaired glucose tolerance or type 2 diabetes than those with normal glucose tolerance, suggesting that Nrg4 may have positive effects on glucose and lipid metabolism (Ma et al., 2016; Tutunchi et al., 2020). The activation of Nrg4 in adipocytes improves metabolic health by increasing adipose tissue angiogenesis (Tutunchi et al., 2020). Thus, it has potential benefits for novel treatments of obesity and associated metabolic complications (Tutunchi et al., 2020).

Different types of exercise training, such as aerobic and resistance training, are associated with benefits such as increased energy expenditure, reduced body fat mass, increased muscle mass, and improvements in insulin resistance and glucose homeostasis in individuals with obesity (Willis et al., 2012; Saeidi et al., 2020b; Zouhal et al., 2020). Frequency, intensity, time, and type of exercise are essential variables in designing an exercise program so as to determine exercise training efficacy and efficiency (Gibson et al., 2018). High-intensity exercise modalities, such as high-intensity interval training (HIIT), are associated with more favorable effects related to increases in post-exercise oxygen consumption (EPOC), body composition variables, and cardiometabolic risk factors in both healthy and individuals with obesity (Batacan et al., 2017; Wewege et al., 2017). Additionally, we recently reported that the intensity of resistance training could determine improvements in body composition, metabolic markers, and inflammatory adipokines in men with obesity (Saeidi et al., 2020b). However, the effects of exercise training and its variables on Nrg4 levels are not well understood. In addition, it is not clear which types of exercise programs are associated with the most efficiency and efficacy in people with obesity.

We speculated that the exercise benefits could be mediated by the activation of Nrg4. To the best of our knowledge, there are no studies on the effects of exercise training on regulating Nrg4 levels in humans. Thus, this study examined two hypotheses: first that exercise training, regardless of modality, positively influences Nrg4 levels, and second, that high-intensity interval training and circuit resistance training increase Nrg4 levels and improve cardiometabolic and body composition markers more than that moderate-intensity continuous training. Therefore, the purpose of this study was to examine the effects of moderate-intensity continuous training, circuit resistance training, and high-intensity interval training on Nrg4 levels, cardiometabolic, and body composition parameters in sedentary males with obesity.

## Materials and Methods

Ninety participants volunteered for the study, 22 (24%) of whom did not meet the following inclusion criteria: BMI > 30 kg/m^2^, being sedentary (no regular physical activity during the last 6 months) and performing only activities of daily living, absent of cardiovascular, metabolic, and endocrine diseases, and not consuming alcohol. Individuals with joint disorders, physical disabilities, and taking prescribed medications and/or supplements that could affect muscle and adipose tissue metabolism were also excluded. Sixty-eight men with obesity (Mean ± SD; age: 27.6 ± 8.4 yrs.; height: 168.4 ± 2.6 cm; weight: 95.7 ± 3.8 kg; BMI: 32.6 ± 2.6 kg/m^2^) voluntarily participated in this study.

All participants completed a physical examination performed by a physician and clinical exercise physiologist on the first visit. All study procedures were explained during this time, and the participants provided a written consent form and Physical Activity Readiness Questionnaire (PAR-Q; Thomas et al., 1992). The Research and Ethics Committee of the University approved all procedures of this study (Ethics code: IR-IAU1398-25). All procedures were performed according to the latest revision of the Declaration of Helsinki.

### Experimental Design

**FIGURE 1:**
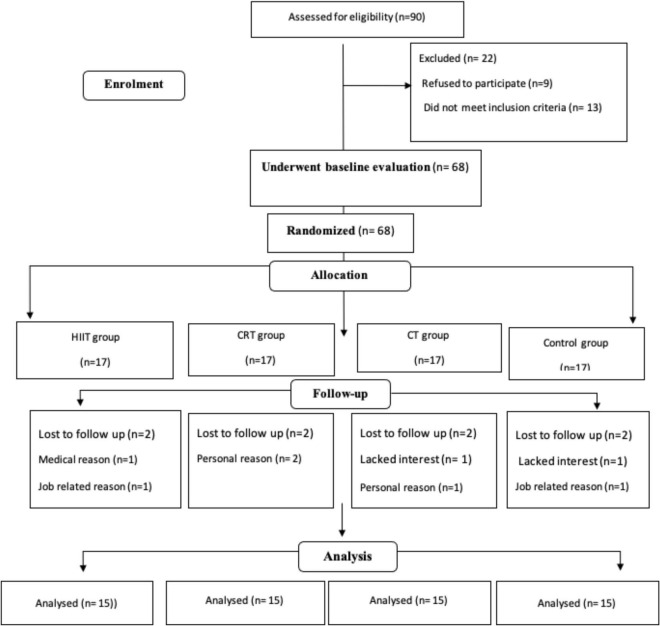
Participant selection flow chart. MICT, Moderate Intensity Continuous Training; CRT, Circuit Resistance Training; and HIIT, High Intensity Interval Training.

All study procedures were explained 1 week prior to the start of the training programs in a participant familiarization session. Height, weight, and body composition were assessed followed by randomly assignment into one of four groups of equal sizes (*n* = 17): Moderate-Intensity Continuous Training (MICT), Circuit Resistance Training (CRT), High-intensity interval training (HIIT), and control groups (Figure 1). Eight participants withdrew from the study due to a variety of reasons (study duration, medical, occupational, and lack of interest in continuing in the study), resulting in 15 participants per group. Instructions on how to perform the training protocols were provided during the third session when body composition variables and VO_2*peak*_ were also measured. Twenty-four hours after the VO_2*peak*_ test, participants in the CRT group performed one maximum repetition test (1-RM) to identify maximum strength based on the Berzisky formula (Brzycki, 1993). Measurements of 1RM and VO_2*peak*_ tests were repeated every 4 weeks. Following baseline measurements, the three training groups (HIIT, CRT, and MICT) started the 12-week exercise training program (3 sessions per week). Participants in the control group were instructed to maintain their current lifestyles until the end of the study. Study measurements were collected at baseline (72 h before the start of the training protocols) and 72 h after the last session in all the groups at the same time of day (within ∼1 h) and under the same environmental conditions (∼20°C and ∼55% humidity). Participants in the training protocols were asked to follow the same diet for 72 h before baseline and before final measurements were obtained.

### Body Composition, Cardio-Respiratory Fitness, and One-Repetition Maximum

Body weights and heights were assessed using a calibrated scale (Seca, Germany) and stadiometer (Seca, Germany), respectively. These data were used to calculate body mass index (BMI) (kg/m^2^). Levels of fat-free mass (FFM), and fat mass (FM) were measured by a bio-impedance analyzer (Medigate Company Inc., Dan-dong Gunpo, South Korea). Evaluation of VO_2*peak*_ was conducted using a modified Bruce protocol (in a temperature-controlled room, 21–23°C) as reported in previous studies of overweight and obese populations (Hunter et al., 2008; Ghroubi et al., 2009), using an electrically motorized treadmill (H/P/Cosmos, Pulsar med 3p- Sports & Medical, Nussdorf-Traunstein Germany). The physiological criteria used to determine VO_2*peak*_ [according to American College of Sports Medicine (ACSM) guidelines] included: if subjects reported they were physically exhausted and reached their maximal effort (according to Borg scale), or if the supervisor recognized the subjects had severe dyspnea, dizziness and other limiting symptoms based on guidelines for CPET test of ACSM and American Heart Association (AHA; Thompson et al., 2010; Myers et al., 2014), with a plateau in VO_2_ and respiratory exchange ratio (RER) ≥ 1.10. Blood pressure was measured with an electronic sphygmomanometer (Kenz BPM AM 300P CE, Japan), and heart rate was monitored with a Polar V800 heart monitor (Finland) throughout the tests. Gas analysis was performed using a gas analyzer system (Metalyzer 3B analyzer, Cortex: biophysik, GMbH, Germany), calibrated before each test. Participants in the CRT protocol lifted their one-repetition maximum (1RM) for 6–8 repetitions for each exercise (see below), in which each 1RM was calculated according to the Berzisky formula (Brzycki, 1993).

### Training Protocols

All exercise training sessions included 7-min warm-up and 7-min cool down at 70% of VO_2*peak*_ and 36 min of a specific exercise training protocol (see below) in the presence of a supervisory exercise physiologist.

The three protocols were matched externally by time (Isotime) and intensity (Isoeffort). All exercise sessions were monitored by heart rate reserve (HRR) which was equalized with the target VO_2*peak*_.

The details of the specific exercises are summarized in Table 1 and described below:

• CRT: 3 sets of 11 circuits (leg extension, knee flexion, sit-ups, chest press, lat pulldown, back extensions, pushups, stationary rowing, biceps curl, military press, and abdominal crunch) with a work-to-rest ratio of 40:20 s and 60 s rest between sets. The load of resistance exercises was 20% of 1RM, and intensity was approximately 70% of VO_2*peak*_ (Kaikkonen et al., 2000).
• HIIT: 6 sets of 3 min of high-intensity exercise at 90% VO_2*peak*_ and 3 min of active recovery at 50% VO_2*peak*_. The average intensity was 70% of VO_2*peak*_ (18 min of high- intensity work, 18 min of active recovery).
• MICT: 36 min of jogging or running on the treadmill at 70% of VO_2*peak*_ (Bartlett et al., 2011).

**TABLE 1:** Exercise protocols by group (MICT, CRT, and HIIT) and by intensity and duration for each weekly session.

12 Weeks	MICT	CRT	HIIT
Session	**Exercises:** treadmill jogging or running. **Time of each session:** 36 min main training +7 min warm-up and 7 min cooldown. **Intensity:** 70% of VO_2*peak*_.	**Exercises:** leg extension, knee flexion, sit ups, chest press, lat pulldown, back extension, pushups, stationary rowing, biceps, military press, and abdominal crunch. **Sets:** 3 Duration of exercise phase: 40 s. **Duration of exercise phase:** 40 s. **Rest interval between exercises:** 20 s. **Rest between each set:** 1 min. **Load:** 20% of 1RM. **Time of each session:** 36 min main training +7 min warm-up and 7 min cooldown. **Intensity:** 70% of VO_2*peak*_.	**Exercises:** treadmill jogging or running. **Time of each session:** 36 min main training +7 min warm-up and 7 min. Sets: 6 **Duration of exercise phase:** 3 min **Exercise intensity:** 90% of VO_2*peak*_. **Duration of recovery phase:** 3 min. **Recovery intensity:** 50% of VO_2*peak*_. ** Mean intensity:** 70% of VO_2*peak*_.

*MICT, Moderate Intensity Continues Training; CRT, Circuit Resistance Training; and HIIT, High Intensity Interval Training.*

Heart rate was monitored every minute throughout the exercise sessions in all training groups using a polar heart rate monitor watch (Polar, Made in Finland). Karvonen’s formula (Camarda et al., 2008) was used to identify HRR to assess exercise intensity in training groups. The range of HRR used in all exercise sessions was between 65 and 75 % HRR. All VO_2*peak*_ measurements were retested, and in the CRT group who performed 1-RM testing, the training intensities were readjusted every 4 weeks.

### Nutrient Intake and Dietary Analysis

Changes in dietary intake were evaluated. Three days of food intake (2 weekdays and 1 weekend day) were recorded before and after 12 weeks. The nutrient intakes were computed using the method of McCance and Widdowson (McCance and Widdowson, 2014). Total energy consumption and the amount of energy derived from proteins, fats, and carbohydrates were also determined (Table 2).

**TABLE 2:** Mean (sd, ±) values of nutritional intake by group.

	Control		MICT		CRT		HIIT	
	Pre	Post	Pre	Post	Pre	Post	Pre	Post
Total energy (kcal/d)	2,225 ± 190	2,306 ± 103	2,254 ± 216	2,274 ± 136	2,203 ± 157	2,287 ± 98	2,188 ± 155	2,283 ± 291
Total protein (g/d)	111 ± 6.30	112 ± 5.73	110 ± 5.47	114 ± 4.53	112 ± 4.13	110 ± 4.43	109 ± 3.50	108 ± 2.63
Protein (g/kg BW/d)	1.05 ± 0.45	1.2 ± 0.4	1.1 ± 0.5	1.2 ± 0.3	1.1 ± 0.4	1.0 ± 0.3	1.0 ± 0.2	1.1 ± 0.3
Total protein (% energy)	18.3 ± 4.1	19.6 ± 3.5	18.1 ± 4.0	19.5 ± 3.0	18.4 ± 4.2	19.7 ± 4.9	18.7 ± 4.4	20.1 ± 5.7
Total CHO (g/d)	295 ± 10.9	304 ± 24.1	305 ± 11.1	316 ± 9.87	285 ± 8.12	289 ± 8.09	241 ± 7.29	244 ± 6.85
Total CHO (% energy)	48.5 ± 6.9	50.8 ± 7.1	50.5 ± 6.3	52.6 ± 7.8	47.4 ± 7.2	50.5 ± 7.0	45.3 ± 9.4	48.0 ± 7.1
Total fat (g/d)	81 ± 5.44	77 ± 7.07	80 ± 5.07	76 ± 5.41	83 ± 5.01	78 ± 4.90	85 ± 6.54	78 ± 5.35
Total fat (% energy)	30.9 ± 7.6	28.8 ± 6.9	31.2 ± 8.8	29.5 ± 7.5	32.5 ± 6.9	29 ± 6.4	34.5 ± 5.9	30.0 ± 6.6

*MICT, Moderate Intensity Continues Training; CRT, Circuit Resistance Training; and HIIT, High Intensity Interval Training.*

### Blood Markers

All testing was carried out under standard conditions between 8 and 10 am. Fasting blood samples were taken from the right arm 12 h and 72 h before the first exercise session and 72 h after the last session. Blood samples were transferred to EDTA-containing tubes, centrifuged for 10 min at 3,000 rpm, and stored at −70°C. Plasma total cholesterol (TC) and triglycerides (TG) were measured by enzymatic methods (CHOD-PAP) using a Pars tech kit (Tehran, Iran) with a coefficient and sensitivity of 1.1% and 5 mg/dl and 1.6% and 5 mg/dl respectively. High-density cholesterol (HDL-C) and low-density cholesterol (LDL-C) levels were determined using a photometric Pars Testem’s Quantitative Detection Kit (Tehran, Iran) with a coefficient and sensitivity of 1.8% and 1 mg/dl and 1.2% and 1 mg/dl, respectively. Very low-density cholesterol (VLDL-C) was calculated using the TG/5 formula (McNamara et al., 1990). Insulin levels were measured with an ELISA kit (Demeditec, Germany) with a sensitivity of 1 ng/ml and the rate of external and internal errors were 5.1 and 8.4%, respectively. Glucose levels were measured with a colorimetric enzymatic kit (Parsazmun, Tehran, Iran) with 5 mg/dl sensitivity. Insulin resistance was assessed using the homeostasis model assessment of insulin resistance (HOMA) according to the formula: HOMA- IR = 22.5 μmol/fasting plasma insulin X fasting plasma glucose (Willis et al., 2012). Nrg4 was measured with an ELISA kit (Kit-ELISA, Germany; Cat number ABIN1571585 with sensitivity of 0.31 ng/ml).

### Statistical Analysis

All data were evaluated with SPSS software (version 22), and the normality of the data was assessed by the Shapiro–Wilk test. One-way ANOVA and Tukey’s *post-hoc* tests were used for evaluation baseline data of four groups. Interactions between groups were determined by a two-way ANOVA repeated measures test (Groups*time). When a significant difference was detected by ANOVA, mean differences were determined by pairwise comparisons. Correlation between Nrg4 levels and other data was measured with Pearson correlation tests. The sample size was calculated to detect a statistical difference between study variables with a 95% confidence interval (CI) and 80% or greater power value. Additionally, effect sizes (ES) were determined from ANOVA output by partial eta-squared. Moreover, within-group ES were computed using the following equation: ES = (mean post-mean pre)/SD (Field, 2013). According to Hopkins et al. (2009) ES are considered trivial (<0.2), small (0.2–0.6), moderate (0.6–1.2), large (1.2–2.0) and very large (2.0–4.0). Descriptive statistics [means, sd (±)] were used to describe all data. A *p*-value of <0.05 was used to indicate statistical significance.

## Results

### Dietary Analysis

No significant differences were observed between the groups in total energy consumption and energy derived from carbohydrate, fat and protein before and after 12 weeks (*p* > 0.05) (Table 2).

### Body Composition Variables and VO_2*peak*_

There were significant interactions between all groups and time for FFM, FM, and VO_2*peak*_ (*p* < 0.05, ES: 0.78, 0.86, 0.59, respectively). Body fat percent decreased in HIIT (−11%), CRT (−8%), and MICT (−8%) protocols compared with the control group (*p* < 0.05) (Table 3). There were increases in FFM following CRT (11%) and HIIT (5%) protocols; however, this increase was significant only in CRT protocol compared with the MICT and control groups (*p* < 0.05) (Table 3). There were increases in VO_2*peak*_ after 12 weeks of HIIT (13%), CRT (10%), and MICT) (7%) compared with the control group (*p* < 0.05) (Table 3). There were non-significant decreases in BMI and body weights following the training programs (*p* > 0.05, ES: 0.13, 0.51, respectively) (Table 3).

**TABLE 3:** Pre and Post mean (sd, ±) cardiometabolic and body composition variables by group.

	Control		MICT		CRT		HIIT	
	Pre	Post	Pre	Post	Pre	Post	Pre	Post
Weight (kg)	94.9 ± 4.62	94.9 ± 4.55	95.9 ± 2.60	93.4 ± 2.47	97.2 ± 3.78	94.4 ± 3.56	95.1 ± 4.27	91.2 ± 3.89
BMI (kg/m^2^)	31.4 ± 1.68	31.6 ± 1.66	31.5 ± 3.59	30.7 ± 1.53	33.7 ± 2.76	32.2 ± 2.57	33.9 ± 2.31	32.2 ± 2.14
FFM (kg)	29.9 ± 1.53	29.5 ± 1.72	29.6 ± 1.44	29.8 ± 1.37	30.0 ± 2.08	33.2 ± 2.12*#	29.4 ± 1.72	31.0 ± 1.13
FM (kg)	32.8 ± 1.85	33.2 ± 1.70	32.1 ± 1.67	29.6 ± 1.68*	32.6 ± 1.95	30.0 ± 1.62*	32.4 ± 1.18	28.8 ± 1.26*
VO_2*peak*_	27.5 ± 2.41	27.2 ± 2.25	29.3 ± 3.26	31.3 ± 2.94*	28.6 ± 2.28	31.6 ± 2.02*	28.4 ± 1.88	32.1 ± 1.76*
HDL (mmol/L)	0.83 ± 0.05	0.83 ± 0.03	0.94 ± 0.03	1.01 ± 0.04*	0.92 ± 0.05	1.23 ± 0.06*#	0.89 ± 0.06	1.17 ± 0.05*#
LDL (mmol/L)	4.08 ± 0.11	4.08 ± 0.10	4.03 ± 0.12	3.75 ± 0.23*	4.06 ± 5.29	3.43 ± 0.13*#	4.13 ± 0.15	3.62 ± 0.11*#
TC (mmol/L)	6.20 ± 0.17	6.33 ± 0.18	6.05 ± 0.15	5.81 ± 0.17*	6.18 ± 0.11	5.63 ± 0.10*	6.20 ± 0.12	5.66 ± 0.11*
TG (mmol/L)	2.69 ± 0.05	2.67 ± 0.05	2.70 ± 0.05	2.66 ± 0.06	2.67 ± 0.06	2.57 ± 0.06*#	2.73 ± 0.06	2.60 ± 0.05
VLDL (mmol/L)	1.22 ± 0.13	1.22 ± 0.11	1.24 ± 0.11	1.22 ± 0.10	1.22 ± 0.13	1.17 ± 0.12	1.25 ± 0.12	1.19 ± 0.11
Glucose (mmol/L)	5.5 ± 0.6	5.7 ± 0.5	5.6 ± 0.5	4.8 ± 0.4*	5.6 ± 0.4	4.6 ± 0.4*	5.7 ± 0.4	4.7 ± 0.4*
Insulin (pmol/L)	110 ± 9.72	113 ± 7.63	110 ± 7.63	100 ± 10.4	109 ± 7.63	87.5 ± 9.7*	108 ± 6.38	86.8 ± 11.1*

MICT, Moderate Intensity Continues Training; CRT, Circuit Resistance Training; HIIT, High Intensity Interval Training; FFM, fat free mass; FM, fat mass; BMI, body mass index; TG, triglyceride; TC, total cholesterol; HDL, high-density lipoprotein; LDL, low-density lipoprotein; and VLDL, very-low-density lipoprotein. * indicates significant differences compared to the control group (p < 0.05). # indicated significant differences between training groups (p < 0.05).

### Lipid Profiles

There were interactions between the exercise training groups and time for HDL, LDL, TC, and TG (*p* < 0.05, ES: 0.83, 0.79, 0.90, 0.52, respectively). Plasma levels of HDL increased in the HIIT, CRT, and MICT protocols compared with the control group (*p* < 0.05) (Table 3). Nevertheless, this increase was more significant in HIIT and CRT protocols compared to the MICT protocol (*p* < 0.05). Levels of LDL and TC decreased in the HIIT and CRT, and MICT protocols compared with control groups (*p* < 0.05) (Table 3). However, LDL levels were reduced in the HIIT and CRT protocols more than in the MICT protocol (*p* < 0.05). Plasma TG levels decreased only in the CRT protocol compared to MICT and control groups (*p* < 0.05). Despite this, VLDL levels were non- significantly decreased in all training protocols (*p* > 0.05, ES: 0.13) (Table 3).

### Insulin, Glucose, and HOMA-IR and Neuregulin 4

**FIGURE 2:**
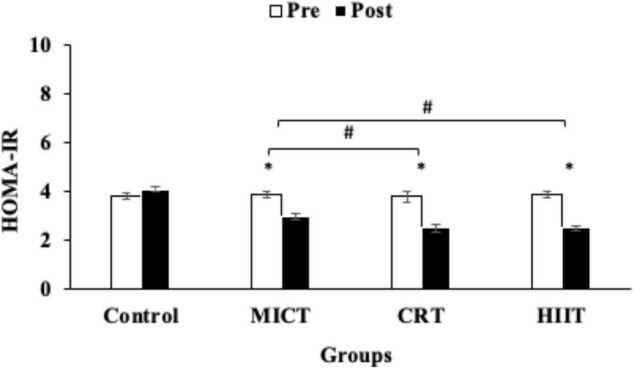
Pre and post training values [mean, sd (±)] for Nrg4 (neuregulin 4) in MICT (Moderate Intensity Continuous Training), CRT (Circuit Resistance Training), and HIIT (High Intensity Interval training) groups. *indicates significant differences from the control group (*P* < 0.05). # indicates significant differences between training groups (*p* < 0.05).

A repeated-measures ANOVA test revealed significant interactions between the exercise groups and time for NRG4, glucose, HOMA-IR, and insulin (*p* < 0.05, ES: 0.98, 0.39, 0.91, 0.61, respectively). Levels of NRG4 were increased in the three training protocols (HIIT, CRT, and MICT) compared with the control group (*p* < 0.05), although this increase was greater in HIIT (160%) and CRT (140%) compared with the MICT protocol (36%) (*p* < 0.05) (Figure 2).

**FIGURE 3:**
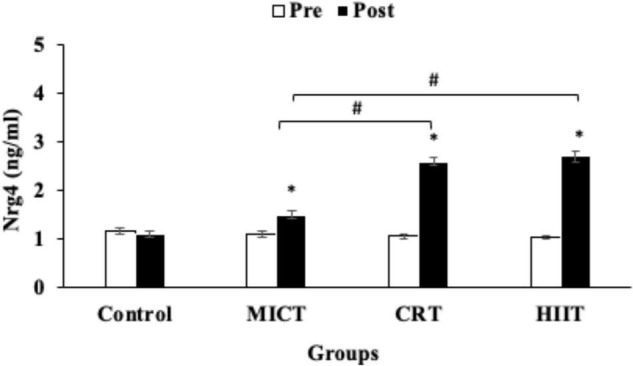
Pre and post training values [mean, sd (±)] for HOMA-IR (Homeostatic Model Assessment-Insulin Resistance), in MICT (Moderate Intensity Continuous Training), CRT (Circuit Resistance Training), and HIIT (High Intensity Interval training) groups. *indicates significant differences from the control group (*P* < 0.05). # indicates significant differences between training groups (*p* < 0.05).

Levels of HOM-IR, and glucose were decreased in the HIIT (−36%, −18%), CRT (−34%, −18%) and MICT (−23%, −15%) protocols compared with the control group (*p* < 0.05) (Figure 3 and Table 3). However, reductions in HOM-IR were greater in CRT and HIIT protocols compared to the MICT protocol (*p* < 0.05) (Figure 3 and Table 3). Plasma levels of insulin were reduced in the decreased following HIIT (−20%), CRT (−20%), and MICT (−9%) protocols, but these reductions were significant only in HIIT and CRT protocols when compared with the control group (*p* < 0.05) (Table 3).

There were significant correlations (*p* < 0.05) between Nrg4 with HDL (*r* = 0.641), LDL (*r* = −0.568), TC (*r* = −0.459), glucose (*r* = −0.691), HOMA-IR (*r* = −0.624), body weight (*r* = −0.655), BMI (*r* = −0.687), FFM (*r* = 0.554), FAT (*r* = −0.674) and VO_2*peak*_ (*r* = 0.745) as determined by Pearson correlations analysis (Table 4). Plasma levels of Nrg4 were not correlated (*p* > 0.05) with TG (*r* = −0.212), VLDL (*r* = −0.195) and insulin (*r* = −0.225) levels (Table 4).

**TABLE 4:** Pearson correlation coefficients between Nrg4 and cardiometabolic and body composition variables.

	Cadiometabolic	Pearson *r*-value	*p*-value
Nrg4 (ng/ml)	HDL (mmol/L)	0.841	*p* < 0.05*
	LDL (mmol/L)	–0.768	*p* < 0.05*
	TC (mmol/L)	–0.759	*p* < 0.05*
	TG (mmol/L)	–0.212	*p* > 0.05
	VLDL (mmol/L)	–0.195	*p* > 0.05
	Glucose (mmol/L)	–0.691	*p* < 0.05*
	Insulin (pmol/L)	–0.225	*p* > 0.05
	HOMA-IR	–0.624	*p* < 0.05*
	Weight (kg)	–0.655	*p* < 0.05*
	BMI (kg/m^2^)	–0.687	*p* < 0.05*
	FFM (kg)	0.754	*p* < 0.05*
	Fat (kg)	–0.674	*p* < 0.05*
	VO_2*peak*_	0.745	*p* < 0.05*

TG, triglyceride; TC, total cholesterol; HDL, high-density lipoprotein; LDL, low-density lipoprotein; VLDL, very-low-density lipoprotein; BMI, body mass index; and FFM, fat free mass. * indicates significant correlation between levels of the measured variable with Nrg4 (neuregulin 4) levels.

## Discussion

We investigated the effects of three different exercise training protocols on Nrg4 levels and cardiometabolic and body composition variables in sedentary men with obesity. Our current study indicates that levels of Nrg4 were increased by the HIIT, CRT, and MICT protocols, with the increases being greater in HIIT and CRT protocols. In addition, the three training protocols decreased HOMA-IR, TC, and LDL levels, but the decreases for LDL and HOM-IR were greater following HIIT and CRT protocols relative to the MICT protocol. Furthermore, insulin and HDL levels were improved only by the CRT and HIIT protocols.

Neuregulin 4 is a member of the epidermal growth factor (EGF) family and is expressed in the lungs, heart, and adipose tissue but most commonly in brown fatty tissue (Pfeifer, 2015). Nrg4 consists of an EGF-like domain that acts as an autocrine/paracrine or endocrine factor after proteolytic breakdown (Pfeifer, 2015). Nrg4 reduces obesity in mice and humans who are also exposed to various metabolic diseases (Chen L. L. et al., 2017; Chen Z. et al., 2017). Many studies have examined the tissue effects of Nrg4, especially in the liver and adipose tissues (Chen Z. et al., 2017; Blüher, 2019), where Nrg4 in fat tissue is reduced by body weight (Wang et al., 2014). Our study indicates that improvement in Nrg4 is accompanied by improvements in HOMA-IR by the MICT, CRT, and HIIT exercise protocols, suggesting a positive correlation of Nrg4 with metabolic status (Wang et al., 2014). Moreover, our study demonstrates that increased levels of Nrg4 were associated with reduced plasma glucose and insulin levels and greater fat percent loss; these findings confirmed our hypothesis and support a previous report that high circulating levels of Nrg4 prevents inflammation, improves insulin resistance, and prevents weight gain (Ma et al., 2016). In addition, low plasma levels of Nrg4 in the blood are independently associated with an increased risk of metabolic syndrome in individuals with obesity, and are also negatively correlated with blood glucose levels and body fat mass (Cai et al., 2016). There are no previous studies on the effects of exercise training on Nrg4 levels in humans that we identified. Our study reports that plasma levels of Nrg4 were increased more significantly by HIIT and CRT protocols, which agree with our original hypothesis. In addition, we report greater reductions of LDL, insulin, and HOMA-IR following HIIT and CRT protocols relative to the MICT protocol, further supporting our hypothesis that exercise intensity regulates Nrg4 levels. Additionally, these results confirm our recent study that showed that resistance training intensity is key in modifying adipokines and metabolic markers in men with obesity (Saeidi et al., 2020b). The greater increases in Nrg4 may be related to higher intensity in these protocols, leading to higher metabolic stress and, consequently, increasing catecholamine and hormone (such as growth hormone and glucagon) levels in an intensity-related manner. The effects of exercise on fat tissue lipolysis are greater in the HIIT and CRT protocols compared to the MICT training protocols (Zouhal et al., 2008; Czech, 2017; Dias et al., 2018; Maillard et al., 2018). Thus improvements in Nrg4 are likely related to greater physiological and metabolic stress cocurring during HIIT and CRT protocols, resulting in more physiological adaptations. These adaptations as reported in this study include greater improvements in body composition variables, HDL, LDL, insulin, and HOM-IR following both HIIT and CRT protocols. However, more studies are needed to clarify the mechanisms for regulating Nrg4 activity by exercise training.

Our study demonstrates reductions of LDL levels in the MICT (−7%), CRT (−15%), and HIIT (−13%) training protocols, with reductions in TC levels of 4, 9, and 9% in the three protocols, respectively. This reduction in LDL in CRT and HIIT protocols was significantly greater relative to MICT protocol, and there was a non-significant decrease in TC in the CRT and HIIT protocols. These results support previous studies on the importance of exercise intensity in adjusting lipid profile (Racil et al., 2013; Blüher et al., 2017). Importantly, our results confirm a possible interaction between Nrg4 and obesity (Little et al., 2014; Ma et al., 2016; Saeidi et al., 2020b), and suggest a positive effect of exercise training on obesity and lipid profiles that may be mediated by Nrg4. In addition, we report increases in HDL following HIIT and CRT protocols. Our study (using three different exercise training protocols) and a previous study report (Thompson et al., 2010) indicate that increased levels of Nrg4 are positively correlated with HDL levels. Other studies reported that high-intensity exercise increases glucose metabolism increases, so decreasing/depleting stores of muscle glycogen; consequently, post-exercise muscle glycogen resynthesis occurs at high levels and possibly stimulates lipid metabolism (Jackson et al., 1988; Gillen et al., 2012). Therefore, it is likely that the improvements in the lipid profile we observed may be related to higher HIIT and CRT intensity, leading to increased lipid metabolism in post-exercise times.

## Study Limitations

Our study is not without limitations. The lifestyles and dietary intakes of participants were not controlled although they were instructed by a nutritionist and were asked to maintain the same diet throughout the study. We used bioelectrical impedance to measure body composition variables and acknowledge that bioelectrical impedance is not a gold standard method for measuring these markers; however its reliability and validity has been reported previously (Jackson et al., 1988; Ling et al., 2011). The protocols used in our study were not equalized according to calorie expenditure, which might have influenced the results. Instead, all protocols were matched by time (Isotime) and intensity (Isoeffort).

## Conclusion

In our study HIIT and CRT caused greater increases in circulating levels of Nrg4 and HDL than those produced by MICT. In addition, both HIIT and CRT also had positive effects on insulin resistance, insulin, and LDL levels. Thus, we conclude that HIIT and CRT may be more useful approaches for reducing metabolic indices associated with cardiovascular disease in sedentary individuals with obesity. However, additional studies are needed to determine the time course response of changes in Nrg4 levels produced by exercise, and analyses are also needed on the effects of HIIT with different rest-to-work ratios and various durations, alone or combined with resistance training.

## Data Availability Statement

The raw data supporting the conclusions of this article will be made available by the authors, without undue reservation.

## Ethics Statement

The studies involving human participants were reviewed and approved by the Research and Ethics Committee of Islamic Azad University (Ethics code: IR-IAU1398-25). The patients/participants provided their written informed consent to participate in this study.

## Author Contributions

FT, NK, SM, AS, and HZ contributed to the conception or design of the work. NK, FT, AS, IL, TM, KJ, SRS, SS, and TV contributed to the data acquisition, analysis, or interpretation. MS, TM, KJ, and PD-B drafted the manuscript. All authors critically revised the manuscript, gave final approval, agreed to be accountable for all aspects of work, ensuring integrity and accuracy, and agreed with the order of presentation of the authors.

## Conflict of Interest

The authors declare that the research was conducted in the absence of any commercial or financial relationships that could be construed as a potential conflict of interest.

## Publisher’s Note

All claims expressed in this article are solely those of the authors and do not necessarily represent those of their affiliated organizations, or those of the publisher, the editors and the reviewers. Any product that may be evaluated in this article, or claim that may be made by its manufacturer, is not guaranteed or endorsed by the publisher.
